# Cancer risk and the complexity of the interactions between environmental and host factors: HENVINET interactive diagrams as simple tools for exploring and understanding the scientific evidence

**DOI:** 10.1186/1476-069X-11-S1-S9

**Published:** 2012-06-28

**Authors:** Domenico F Merlo, Rosangela Filiberti, Michael Kobernus, Alena Bartonova, Marija Gamulin, Zeljko Ferencic, Maria Dusinska, Aleksandra Fucic

**Affiliations:** 1 IRCCS AOU San Martino-IST-National Cancer Research Institute, Genoa, Italy; 2 NILU – Norwegian Institute for Air Research, Kjeller, Norway; 3 University Hospital “Zagreb”, Zagreb, Croatia; 4 Children’s Hospital “Srebrnjak”, Zagreb, Croatia; 5 Slovak Medical University, Bratislava, Slovakia; 6 Institute for Medical Research and Occupational Health, Zagreb, Croatia

## Abstract

**Background:**

Development of graphical/visual presentations of cancer etiology caused by environmental stressors is a process that requires combining the complex biological interactions between xenobiotics in living and occupational environment with genes (gene-environment interaction) and genomic and non-genomic based disease specific mechanisms in living organisms. Traditionally, presentation of causal relationships includes the statistical association between exposure to one xenobiotic and the disease corrected for the effect of potential confounders.

**Methods:**

Within the FP6 project HENVINET, we aimed at considering together all known agents and mechanisms involved in development of selected cancer types. Selection of cancer types for causal diagrams was based on the corpus of available data and reported relative risk (RR). In constructing causal diagrams the complexity of the interactions between xenobiotics was considered a priority in the interpretation of cancer risk. Additionally, gene-environment interactions were incorporated such as polymorphisms in genes for repair and for phase I and II enzymes involved in metabolism of xenobiotics and their elimination. Information on possible age or gender susceptibility is also included. Diagrams are user friendly thanks to multistep access to information packages and the possibility of referring to related literature and a glossary of terms. Diagrams cover both chemical and physical agents (ionizing and non-ionizing radiation) and provide basic information on the strength of the association between type of exposure and cancer risk reported by human studies and supported by mechanistic studies. Causal diagrams developed within HENVINET project represent a valuable source of information for professionals working in the field of environmental health and epidemiology, and as educational material for students.

**Introduction:**

Cancer risk results from a complex interaction of environmental exposures with inherited gene polymorphisms, genetic burden collected during development and non genomic capacity of response to environmental insults. In order to adopt effective preventive measures and the associated regulatory actions, a comprehensive investigation of cancer etiology is crucial. Variations and fluctuations of cancer incidence in human populations do not necessarily reflect environmental pollution policies or population distribution of polymorphisms of genes known to be associated with increased cancer risk. Tools which may be used in such a comprehensive research, including molecular biology applied to field studies, require a methodological shift from the reductionism that has been used until recently as a basic axiom in interpretation of data. The complexity of the interactions between cells, genes and the environment, i.e. the resonance of the living matter with the environment, can be synthesized by systems biology. Within the HENVINET project such philosophy was followed in order to develop interactive causal diagrams for the investigation of cancers with possible etiology in environmental exposure.

**Results:**

Causal diagrams represent integrated knowledge and seed tool for their future development and development of similar diagrams for other environmentally related diseases such as asthma or sterility. In this paper development and application of causal diagrams for cancer are presented and discussed.

## Background

### Cancer incidence and mortality

**Figure 1:**
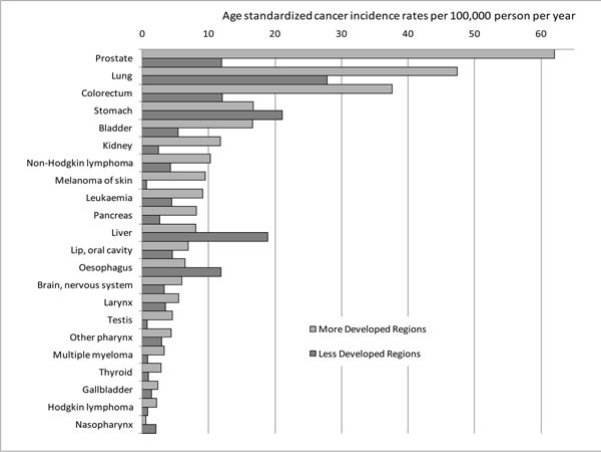
**Cancer incidence rates in males (upper panel) and females (lower panel) in more and less developed regions worldwide** Source: [1]

The estimated global burden of cancer amounts to some 12,667,400 new cancer cases worldwide in 2008 [1]. Colorectal, lung, breast, prostate, stomach and liver cancer are the most frequently diagnosed cancers. Stomach, liver, oesophageal and cervical cancers incidence rates are higher in populations living in less developed regions (Figure 1) than in more developed regions [1]. These data show the significant role played by socioeconomic status in cancer risk.

**Figure 2:**
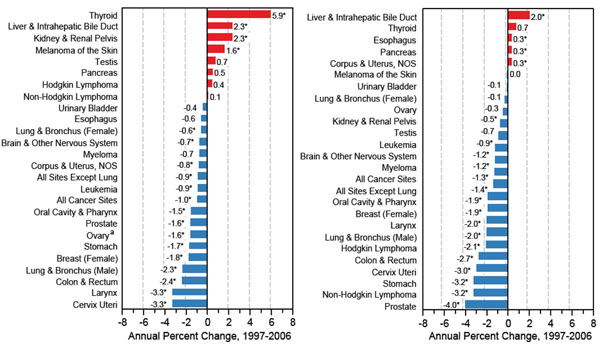
**Trends in SEER incidence (left panel) and US mortality rates (right panel) by cancer site, years 1997-2006** Rates per 100,000, age-adjusted to the 2000 US standard population. Source: [25]

**Figure 3:**
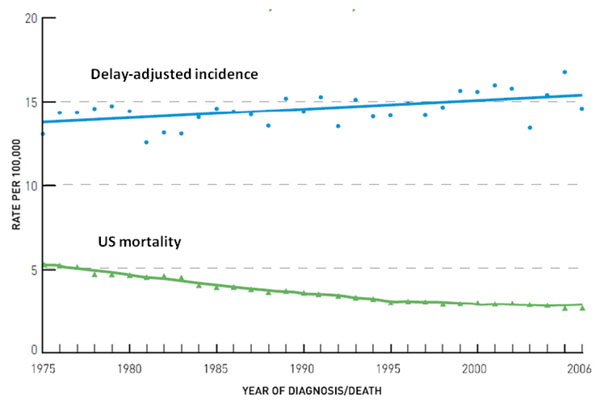
**Delay-adjusted incidence and U.S. mortality trends: all childhood cancers, <20 years of age at diagnosis, both sexes and all races during the time period 1975–2006** Rates are age-adjusted to the 2000 U.S. standard population. Delay-adjusted incidence is an algorithm used to estimate incidence if it were unaffected by reporting delays. Source: [25]

**Figure 4:**
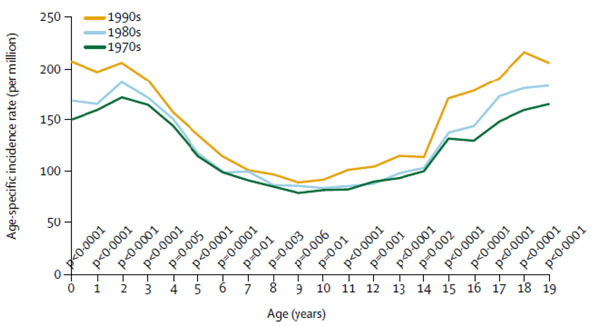
**Age specific cancer incidence trends among European children and adolescent (<20 years old) during the time period 1970–1999** P values test difference between first and last decade. Source: [5]

Trends in cancer incidence between mid 1990s and early 2000 decreased in Northern and Western European countries with the exception of obesity related cancers. Although a decreased incidence and mortality was detected for tobacco-related cancers (i.e., cancers of the lung, larynx, and oesophagus) for males in Northern, Western and Southern Europe, increased rates were observed among females nearly everywhere in Europe and for both sexes in central European regions. The estimated annual percentage change for lung cancer in men ranged between -0.4% and -4% while among women the observed increase ranged between 0.6% and 5% [2]. During the decade from 1997 to 2006, cancer incidence decreased in the United States by an average of 1 percent per year and overall cancer mortality declined also (Figure 2) [3]. The decline of death rates was bigger for men than women. Lung, prostate and colorectal cancers in men and breast and colorectal cancers in women, the most frequently occurring cancers, were responsible for the observed decline. Despite the observed reduction, increased incidence rates were found among men for cancers of the liver, kidney and oesophagus, and for melanoma and myeloma, and, among women, for cancers of the lung, thyroid, pancreas, brain and nervous system, bladder and kidney, and for melanoma. Rates of leukaemia and non-Hodgkin’s lymphoma increased in both sexes. Some 1,479,350 cases are expected to be diagnosed in 2009, excluding non invasive cancer (carcinoma in situ) of any site except urinary bladder, and basal and squamous cell skin cancers (the latter are expected to be about 1 million cases). The probability of developing an invasive cancer for a male US citizen is 1.42 (1 in 70) from birth to the age of 39 and 43.89 (1 in 2) from birth to death. For a female US citizen is 2.07 (1 in 48) from birth to the age of 39 and 37.35 (1 in 3) from birth to death [4]. Cancer incidence increased during the same period among US children (Figure 3) [3] and amongst European children and adolescents during the period 1970–99 (Figure 4) [5]. For most cancer types incidence increased by 1·0% per year among European children (< 15 years old) and by 1·5% in adolescents (15–19 years).

### Environmental exposure complexity and need for action

Environmental exposures may modulate a variety of biologic processes such as gene expression and gene repair mechanisms, hormone production/function, and inflammation [6,7].

Moreover, the delayed adverse health effects of exposures occurring during critical windows of vulnerability (e.g., early life, including the prenatal period and puberty) remain largely unknown. One well known exception is in utero exposure to diethylstilbestrol (DES) which increases the risk of benign and malignant pathology in the third generation [8]. Other agents such as ambient air PAHs and PM2.5 have been shown to influence maturation of the immune system during gestation via shifts in cord blood lymphocytes distributions [9,10]. Whether these shifts will affect cancer risk (or other adverse health outcomes) later in life, need to be proven [11].

Providing undisputable evidence that environmental exposure to complex mixtures of pollutants results in increased cancer risk is challenging for human epidemiologic and experimental studies conducted in vitro and in laboratory animals. Environmental epidemiology, despite its observational nature, is the scientific discipline attempting to make conclusions on disease etiology in human beings. Experimental studies, conducted under controlled conditions, provide “proof of action” of a given exposure in selected biological (e.g., cell culture) or animal models. The two types of scientific evidence are combined together by the scientific community to classify exposures as carcinogenic, probably/possibly or non carcinogenic to humans. Based on the evidence of carcinogenicity, governments and regulatory agencies should establish and implement effective regulation of environmental exposure. Current regulatory approach is of a reactive type (i.e., human harm must be proven before any action is taken). However, some 80,000 chemicals are in use today and 1,000-2,000 new chemicals are synthesized and enter the environment each year, a figure that is impressive especially if one considers that such chemicals may interact with each others, with physical agents, viruses, and thousands of natural compounds [12]. Cancer incidence reflects lifetime exposure to man-made and naturally occurring carcinogens that are present in the living environment. Most of the evidence of the role played by environmental carcinogens has accumulated during the last century [13]. Epidemiologic and animal studies significantly contributed to the discovery of the major causes of cancer and nowadays it is accepted that cancer risk is connected to the living environment through complex interactions between exposures and host factors, the former playing a major role in cancer development. Host factors, such as single-gene inherited cancer syndrome and the polymorphic distribution of genes for cellular detoxification and DNA-repair processes are known to account for a small proportion of the cancer burden in human populations. A large proportion of cancers are believed to be the consequence of multiple exposures that occur over years or persist for a lifetime [14]. There is also evidence that cancer susceptibility resulting from environmental exposures may be inherited by a child when a carcinogen causes germ cell genetic damage in exposed parents [15,16].

Despite the fact that our knowledge of the biologic mechanisms underlying cancer development has been extensively improved, the mechanisms by which environmental contaminants contribute to cancer risk, and particularly how they interact, remain largely under investigated in humans [14].

Is it waiting for a “proof of harm” the right approach to protect human health by reducing exposure? The USA President’s Cancer Panel [14] assessed the state of environmental research on cancer, policy and programs receiving testimony from 45 invited experts from academia, government, industry, the environmental and cancer communities, and the public. The Panel made recommendations for policy, research, program, industry, and other actions aimed at minimizing the impact of environmental factors on cancer. A precautionary oriented approach instead of the reactionary approach currently used is recommended by the President’s Cancer Panel as the cornerstone of a new cancer prevention strategy based on primary prevention. Such a recommended approach should “shift the burden of proving safety to manufacturers prior to new chemical approval, in mandatory post-market studies for new and existing agents, and in renewal applications for chemical approval”. The European Commission has anticipated, to some extent, the US by adopting in 2007 a precautionary approach to chemical regulation. The Registration, Evaluation, Authorization, and Restriction of Chemical Substances (REACH)[17] is a major reform that requires industry to take a main role in managing risks from chemicals by providing safety information on its products. The final goal of REACH is to protect human health as well as the environment through better and earlier recognition of intrinsic properties of chemicals.

## Methods

### The HENVINET approach to environmental cancers

The Health and Environment Network (HENVINET) was funded by the Commission of the European Communities within the 6th Framework Programme on Research, Technological Development and Demonstration. The main objective of HENVINET was that of establishing a long-term co-operation between researchers, policy makers and stakeholders in the area of environment and health research and assessment. To protect the health of populations and individuals, environmental and health policies need to integrate environmental and health knowledge: HENVINET is meant to support such informed policy making process. Based on the four priority health diseases of the European Environment and Health Action Plan 2004-2010 (EHAP)[18] (i.e., asthma and allergies, cancer, neurodevelopmental disorders and endocrine disrupting effects), HENVINET has reviewed, exploited and disseminated knowledge on environmental health issues [18]. EHAP is aimed at improving the health of European citizens, a goal requiring knowing exactly what impact environmental damage has on human health. EHAP was designed to provide the European Union (EU) with reliable information on that impact and to step up cooperation between stakeholders in the environment, health and research fields.

**Figure 5:**
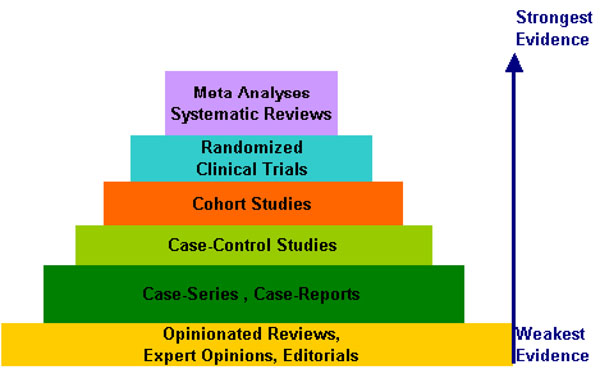
Framework for environment and cancer: strength of the evidence from different study designs

**Figure 6:**
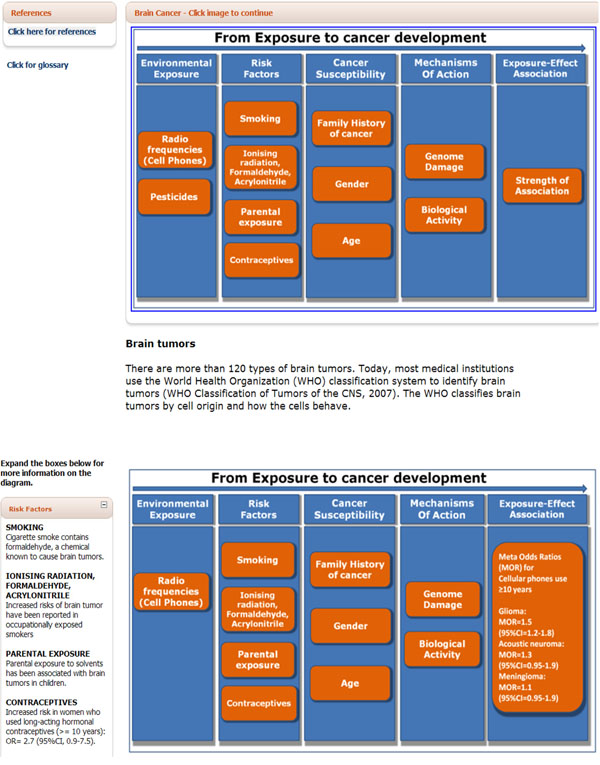
Appearance of the interactive diagrams in the HENVINET portal http://www.henvinet.eu/

The identification of environmental causes of cancer is among the major thrusts of cancer and carcinogenesis research. The systematic review of the epidemiologic evidence available has been used as a tool for the evaluation of the exposure-effect association (causal association) in human studies. Scientific evidence comes from different epidemiologic study designs (Figure 6), of which some are considered to provide a stronger level of evidence than others. Based on their inherent characteristics, their hierarchy is graphically summarized in a pyramid (Figure 5). The pyramid depicts the strength of the evidence for commonly used designs. Such hierarchy should be taken into account in evaluating the published evidence. Therefore, HENVINET reviews on environmental exposure and cancer risk in human populations prioritized the available systematic reviews (i.e., meta-analysis). The elective epidemiologic measure of associations was the meta-relative risk or its estimates (meta-odds ratio, meta-rate ratios, etc.). In addition, the relevant biological aspects /mechanisms underlying cancer development reported by experimental studies were considered as proof of action supporting the epidemiological evidence. All this information was retrieved, scrutinized and summarized in the form of interactive cause-effect diagrams showing, in a simple fashion, the associations between complex environmental exposure and cancer development considered within the HENVINET project.

## Results

### HENVINET interactive cause-effect diagrams

Cancer is not a single disease and cancer risk results from exposure to complex environmental settings (i.e., different exposures) jointly contributing to cancer development. In addition to the environmental risk factors, individual and genetic based susceptibility factors, known as host factors, are playing a role in the process of human carcinogenesis, acting as effect modifiers, which need to be included in the causal framework. The development of interactive diagrams is challenged by the need to provide a summary of the evidences of the exposure-effect associations while accounting for the complexity of the biological and statistical relationships detected along the path leading to cancer development and diagnosis. Environmental exposure(s), known risk factors (e.g., smoking and drinking habits, age, gender), including individual susceptibility (e.g., genetic polymorphisms, family history of cancer) known to be of relevance in terms of differential risk were reported in the diagrams to provide an overview of the continuum from exposure to cancer development. Diagrams included also the evidence from experimental studies when providing a “proof of action” of the environmental exposures considered (i.e., mechanisms of action) and the estimated epidemiologic measure of effect (e.g., meta-relative risk estimates, relative risk, odds ratio). The cancer types and environmental exposures considered within HENVINET are reported in Table 1.

**Table 1:** List of cancers and environmental exposure considered within HENVINET

Cancer types	Environmental exposures
Breast Cancer	Alcohol
	DDT and DDE
	PCB
	PAHs
	
Lung cancer and	Arsenic
Malignant Mesothelioma	Asbestos
	PM2.5
	Radon
	
Brain Tumors	Radiofrequency
	Pesticides
	
Colorectal Cancer	Meat consumption
	Fruits and vegetables consumption
	Intake of calcium and Vitamin D
	Intake of folic acid
	
Leukemia	Low frequency electromagnetic fields
	Pesticides
	Low level ionizing radiation
	
Melanoma	UV light, artificial light
	Ionizing radiation
	Cosmetics (including sun screen)
	Photosensitizing drugs
	Exogenous hormones

### Glossary and references

A glossary and selected references are made available to users to ensure fluent browsing and transparency. The glossary is an important tool aimed at assuring a consistent terminology across the exposure-cancer diagrams (e.g., meaning of reported biological effects, biological activity). Diagrams were specifically developed to allow users to actively explore the depicted exposure-effect interactions within the continuum between cancer initiation and detection. Their appearance is shown in Figure 6 as an example for exposure to radiofrequency and its association with brain tumours, one the hottest and most controversial topic in environmental health. The reader, after selecting a specific environmental exposure within a given cancer type, access a diagram showing the known risk factors, the evidence of susceptibility available, the reported mechanisms of action for a given environmental exposure/agent, and the quantitative measure of the exposure-effect association estimated by recent systematic review. The glossary and the reference list can be both accessed through a link placed on the left side of the interactive diagrams accessible on the HENVINET portal (Figure 6).

### Evaluation of knowledge: the online questionnaires

**Figure 7:**
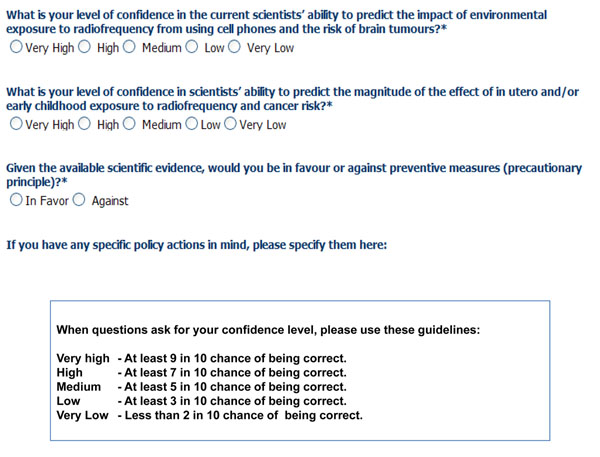
Questionnaire items used for the evaluation of knowledge (questions for radiofrequency and brain cancer)

For each interactive diagram a questionnaire including a limited set of items (questionnaire) was prepared to allow expert reviewers and users to express their level of confidence on the current scientific evidence and the understanding of the various aspects of the exposure-cancer diagram examined. 13 expert reviewers included researchers from the following fields: environmental and occupational epidemiology, cancer epidemiology, risk assessment, exposure assessment, molecular/biomarkers epidemiology, medical statistics, and atmospheric pollution and health effects. Nine of them accepted to review the interactive diagrams and filled in the questionnaire. The structure of the questionnaires has been standardized to provide similar questions across the paths of the exposure-adverse effects considered within the project (i.e., asthma and allergies, cancer, neurodevelopmental disorders, and endocrine disruptors). For each question included in the questionnaires the level of confidence was scored by expert reviewers as very high, high, medium, low and very low. An example of the questions included in the questionnaire for the association between exposure to radiofrequency and brain tumours is shown in Figure 7. The causal diagrams were made accessible to experts selected according to their experience in environmental health and/or oncology. This process is identified in the HENVINET portal as the evaluation of knowledge. Review of diagrams performed by experts was also an exercise for testing of the questionnaire which is meant to be used by readers with different background.

### Causal diagram evaluation

HENVINET cancer causal diagrams were actually a new experience for experts as they offer a simultaneous overview of all xenobiotics described in the etiology of selected site specific cancers.

**Figure 8:**
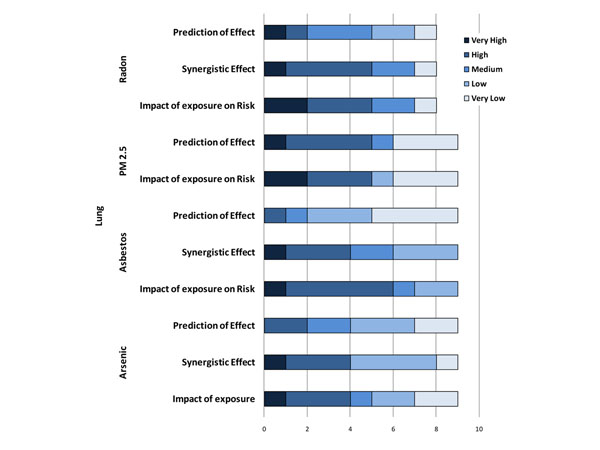
Level of confidence declared by expert reviewers on the scientific evidence reported for selected environmental exposures on their predictive role, synergistic effect, individual susceptibility and lung cancer risk

**Figure 9:**
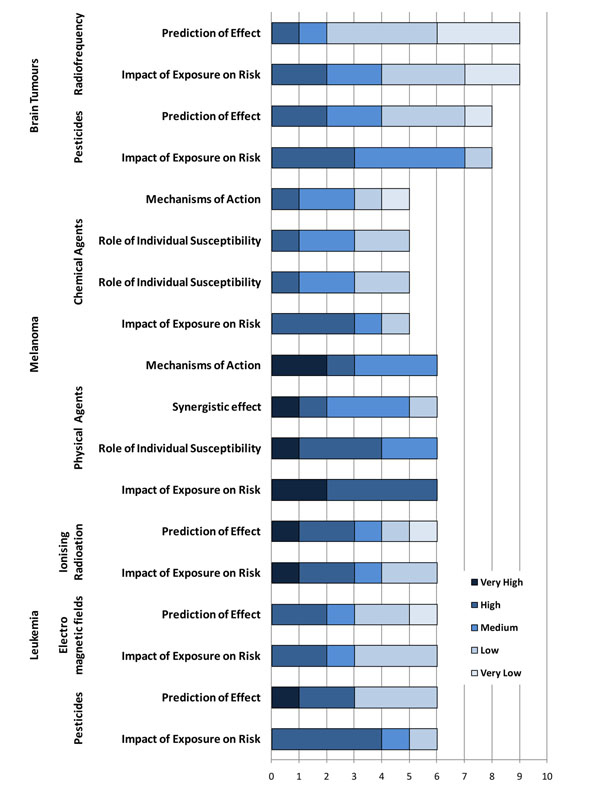
Level of confidence declared by expert reviewers on the scientific evidence reported for selected environmental exposures on their predictive role, synergistic effect, individual susceptibility and the risk of brain tumours, melanoma and leukaemia

**Figure 10:**
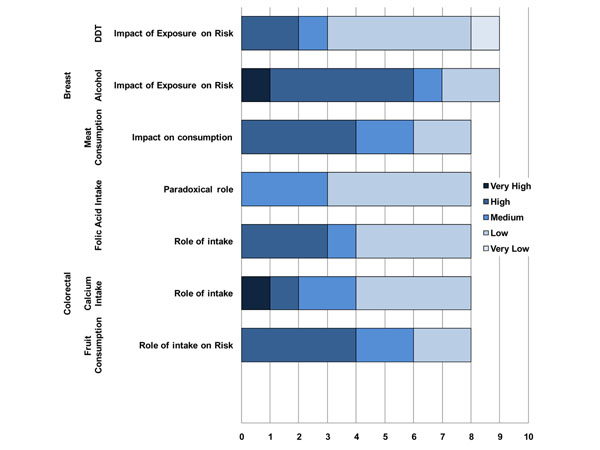
Level of confidence declared by expert reviewers on the scientific evidence reported for selected environmental exposures on their predictive role, synergistic effect, individual susceptibility and the risk of breast and colorectal cancers.

Based on the assumption that expert reviewers should be able to come to exact agreement about how to apply the possible five levels of scoring to each questions, consensus indexes of interpreter reliability were computed as estimates of how experts shared a common interpretation of the construct. The consensus index ranges between 1 (full agreement) and 0 (no agreement). An unexpectedly low consensus index was detected for the questions related to the role of exposure to environmental level of arsenic ( 0.43), radon (0.54), and PM2.5 (0.27) on lung cancer risk and for polycyclic aromatic hydrocarbons (PAH) on breast cancer (0.51) (Figure 8a-c). While for arsenic in drinking water and airborne PM2.5 the causal association with lung cancer is still subjects of controversy, there is clear evidence of a link between indoor radon and lung cancer risk. Indeed, residential radon is recognized as an important cause of lung cancer in the general population with an excess risk of 10% per 100 Bq m3 [19-24]. The generally low agreement between expert reviewers raises the need for better knowledge communication and inclusion of other media for knowledge dissemination. The highest consensus was reached for questions regarding melanoma, especially as far as the role played by physical agents (0.83), pesticides and leukaemia (0.73) and brain tumours (0.77). The experts have a high to very high level of confidence in the scientists' abilities to predict the impact of exposure and individual susceptibilities to physical agents (0.77) on melanoma cancer risk. The results of the evaluations are graphically presented in Figures 8-10.

**Figure 11:**
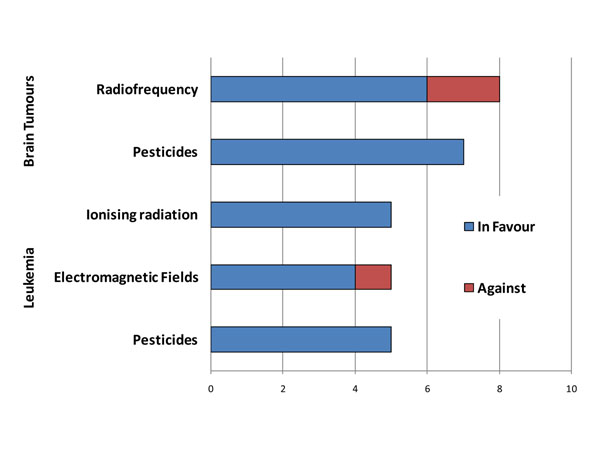
Expert reviewers’ position based on the scientific evidence available concerning the need for precautionary policies for selected environmental exposures

The position of the expert reviewers on the scientific evidence based justification for precautionary policies aimed at containing environmental exposures to electromagnetic and radiofrequency fields and pesticides reported to be associated with brain tumours and leukaemia is shown in Figure 11. All reviewers agreed on the need for precautionary policies for pesticides and ionizing radiation while the consensus on the need for precautionary policies was lower for radiofrequencies and power lines electromagnetic fields.

Discrepancy between the state of the art (e.g. existing knowledge) and answers of experts shows that knowledge is not equally communicated to different professional areas, policy makers and the general public.

## Discussion and conclusions

### How to develop knowledge communication and self-learning interaction between science and policymaking system?

The complexity of the causal relations between exposure to environmental agents, their interactions, as well as the role played by host factors such as age at exposure (e.g., in utero exposure), gender, and polymorphisms of genes involved in the activation-detoxification of xenobiotics, cell cycle, in DNA repair and apoptosis, is not taken into account in current legislation. Environmental health regulatory policies should adopt a new approach which includes the knowledge of complexity. HENVINET interactive causal diagrams are an opportunity for collaboration between the scientific and regulatory communities and the society with its variety of populations, cultures, and environmental differences. The analysis of causal diagrams and the development of specific web sites which enable such an opportunity for an interactive dialogue may represent a starting point for accomplishing effective legislation aimed at protecting health and the environment. Policymakers have to learn the potential of present knowledge and timely deal with the scientific evidence generated by human and laboratory studies that investigate early health effects and or molecular markers that occur and can be measured along the pathways from exposure to disease manifestation. Within this modern and highly technological research framework a precautionary approach can be applied to environment and health issues (not just as an alternative to cost-benefit analysis), with the aim of improving future legislation. The recognition of environmental threats and the prediction of possible associated cancer risks will allow putting scientific facts directly in a regulatory perspective, raise public confidence in science and administration.

## Competing interests

None of the authors have conflict of interest to declare.

## Authors' contributions

All authors contributed equally to the conduct of the research and the writing of the manuscript. DFM has leaded the work and drafted the manuscript. AF was co-responsible for all aspects of the study. RF, MG and ZF participated in the design, implementation and interpretation. MD participated in the design and interpretation. MK has been responsible for the web application and data management. AB coordinated the project and contributed to the concept and interpretation of the results. All authors have read and approved the manuscript.
